# Pulmonary Consumption

**Published:** 1835

**Authors:** 


### Art. IX.—Pulmonary Consumption.

*A Treatise on Pulmonary Consumption, comprehending an inquiry into the causes, nature, prevention, and treatment of Tuberculous and Scrophulous Diseases in general. By James Clark, M. D., F. R. S., Consulting Physician to the King and Queen of the Belgians, and Physician in ordinary to their Royal Highness, the Duchess of Kent and the Princess Victoria.—Philadelphia: Carey, Lea, and Blanchard*—1835. p. p. 296. By the Jun. Editor.

No subject in pathology, has received more attention, for the last twenty years, than lesions of the lungs; and few, perhaps, at the present period, are better understood. The researches of Laennec have done more in pulmonary disease than those of any other, notwithstanding Andral and Louis have, by their discriminating minds and extensive observations, given a precision to the subject of tubercular disease, previously unknown even to this able writer; still they have, in general, confirmed, rather than altered, the opinions of their countryman.

Broussais, amidst his varied fantasies of exclusive solidism, attributed the formation of tubercles entirely to a local inflammation, and it is probable that this opinion is still prevalent, to a certain extent, at least among his followers. The most careless observer must be aware, however, that inflammation of all the tissues of the lungs is frequently present, without leaving a single trace of tuberculous secretion. It is true, inflammation sometimes appears to develope tubercles in scrophulous habits, still it is not certain that they did not exist, to some extent, long before the inflammation occurred. It may also arouse a previous predisposition to this peculiar secretion, which might have remained inactive, for a longer or shorter time, had no such irritation superseded. The distinguished men already named, have, to a great extent however, dissipated the mists of error which, but too frequently, enveloped the pathology of Broussais, and proved, beyond a doubt, that tubercles may exist in the lungs, where no traces whatever are to be found of previous inflammation. A knowledge of this fact is of much importance in the treatment of pulmonary diseases, for where consumption and inflammation of the lungs, acute or chronic, are considered identical, or related as cause and effect, many individuals afflicted with thoracic diseases, are abandoned as utterly incurable, while a judicious treatment might have saved them from an untimely grave.

Our author does not consider tuberculous lesions, whether in the lungs or elsewhere, as local, but affirms that they have their origin either in some hereditary taint, or a morbid state of the constitution, induced by various foreign causes. This, undoubtedly, is the true pathology of the disease, for tubercles, according to Louis and Andral, are seldom found, to any extent, in the lungs, without being also present in some of the abdominal or other vicera. He believes, that true phthisis may often be prevented, but never, or but very seldom cured, even in the incipient stages. After the disease is fairly established, he thinks it would be quite as impossible to cure it, as to restore vision after the eye was destroyed or the functions of the brain, after it had been reduced to a pultaceous mass. The only hopes of saving the individual predisposed to consumption, is to prevent the formation of tubercules in the system, by a judicious treatment. Our author regards true tuberculous phthisis as almost exclusively hereditary, and seldom arising from any inflammatory lesion of the thoracic organs. We do not know what differences there may be between the constitutions of Europeans and Americans, but it is certain, that while the greater portion of the consumptions of our great valley may originate in some vice of nutrition which is hereditary, still a large number of cases depend upon secondary causes, and hence we should persevere in our efforts to cure our patients, especially as it is extremely difficult to draw the distinction between *curable* chronic inflammation of the lungs, and *incurable* tubercular consumption.

The amount of tubercular disease in Great Britain and Ireland is certainly alarming. A few years since, Dr. Young and some others, inferred from the bills of mortality that one-fourth of all the deaths occurring in both, were the offspring of consumption; but our author supposes that this estimate is too low, and proposes one-third, as the proper amount. This is certainly too high for the United States, at least for the Valley of the Mississippi; for it is probable, that consumption does not occasion more than one-eighth, at furthest, of the deaths which annually take place in this wide extent of country.

According to the observations of Dr. Clark, consumption is on the increase among the middle and upper classes of society, in Great Britain. This is probably the result of their modes of living, together with the mental excitement to which they are occasionally subject. If the increase of disease should be rapid, or if it is transferred from parent to offspring, the time will eventually arrive when the aristocracy will disappear. This will take place earlier in England, than in those countries where all classes of society mingle, in the course of a few generations, by marriage.

Our author designates the particular morbid condition of the system, which gives rise to the deposition of tuberculous matter, upon the application of slight exciting causes, *tuberculous cachexia.* This state, he observes, may exist from birth, or may be acquired at almost every period of life. When hereditary, it is manifested by a peculiar modification of the whole organization, which he describes as follows: p. 18.

"The countenance generally affords strong indications of the presence of this affection; in early childhood it has a pale, pasty appearance, the cheeks are generally full, and the upper lip and nose tumid. If the complexion be dark, the color of the skin is generally sallow; if fair, it has an unnatural white appearance, resembling blanched wax rather than healthy integument; and the veins are large and conspicuous. At a more advanced period of youth, the character of the constitution is still more clearly indicated by the countenance. The eyes, particularly the pupils, are generally large, the eyelashes long; and there is usually a placid expression, often great beauty of countenance, especially in persons of a fair florid complexion. On the other hand, in those of a dark complexion, the features are generally less regular, and the skin is commonly coarse, and of a sallow, dingy hue; although there are many exceptions to this, in the fine dark eye, regular features, and delicate skin of such persons. But it is far more easy to distinguish than to describe with accuracy the tuberculous physiognomy, as it varies in every intermediate shade, between the pale, faded, but changing color of persons little under the influence of this morbid condition, and the peculiar permanent sallow cast of countenance which attends the confirmed cachectic state.

In early infancy there is little remarkable in the form of the body; it is generally large, but has not the firmness of health. As the child increases in age, we find that the different parts are not well proportioned, and there is a want of symmetry in the whole. The head is often large, the trunk small, the abdomen tubid, and the limbs are unshapely, being either large and clumsy, or disproportionately slender with large joints: but this is only the case in the more perfect examples of hereditary tuberculous cachexia. The growth of the body is also generally unsteady in its progress; very often it is slowly and imperfectly developed; in other cases it is unusually rapid, particularly towards puberty.

The physical powers are generally below the usual standard. The limbs, though full, are soft, and have neither the form nor the firmness of health. The circulation is generally feeble, as is indicated by a weak pulse and cold extremities. This state of the circulating system forms, I am disposed to believe, an element in the tuberculous constitution; at least I have rarely found it wanting, and regard it as affording an explanation of many of the phenomena of the disease. A full development of the body and great muscular power are not, however, incompatible with a degree of tuberculous cachexia. Several of our celebrated pugilists have died tuberculous. Independently of their bearing on the present subject, such examples deserve attention, and showing the effect of training in increasing the strength even of the tuberculous system.

The functions of organic life are all more or less imperfectly performed, but those more immediately connected with nutrition, particularly the digestive function, are most evidently deranged. Digestion is rarely well performed; the bowels are irregular, more frequently slow in their action than the reverse, and the evacuations have not the appearance which they are known to possess in health. The urinary secretion, also, deviates from the healthy characters, being commonly turbid, particularly when the bowels are costive.

The cutaneous functions are very generally in an unhealthy state; the skin is either soft and flaccid, or dry and harsh, and chronic eruptions of the dry, scaly character, are frequent. In general the insensible perspiration is defective, although copious partial perspirations are common, particularly on the feet, where they often have a fetid odor. But of all these disordered functions, that which claims our principal attention, because it is the primary one, and from it arise most of the others, is the disorder of the digestive organs. The dyspepsia of the tuberculous constitution has peculiar characters by which it may be generally known."

Our author does not think that consumption is connected with any special temperament. He says all are equally obnoxious to it. The strumous dyspepsia of Dr. Todd, which is often the cause of the disease, also attends it in any or all of the temperaments. This affection of the bowels, in children especially, is characterized by the following symptoms, which cannot be too strictly studied. p. 21.

The tongue is redder than natural, especially towards the extremity and along the margins; the anterior part is thickly spotted with small red points of a still brighter color, the central portion being more or less furred, in general according to the duration of the disorder; sometimes the tongue is covered with a dirty whitish fur, through which the red papillæ project; in which case the central part of the tongue is often dry and of a brownish color in the morning. There is generally some thirst: the appetite is variable, more frequently craving than deficient, seldom natural; and the breath is fetid. The bowels are generally confined, though occasionally they are too loose; the evacuations are always unnatural, generally of a pale, greyish colour, of the consistence and appearance of moist clay; and they are often mixed with mocus and partially digested food. The urine is often turbid, sometimes high-colored, and at other times too abundant and pale. The skin is generally harsh and dry, or subject to cold perspirations, particularly the hands and feet, which are habitually cold; copious partial night-sweats are common. The sleep is seldom sound,—the child is restless, and talks in his sleep, or grinds his teeth.

When this disordered state has continued for some time, the complexion loses its natural color, and the face has a pasty, faded aspect; the child is languid, disinclined for exercise or play, and generally fretful; a train of secondary disorders, also soon appears, as a consequence of the irritation of the digestive organs. The internal fauces become full and red, inflammatory sore-throats are common, and the tonsils often become permanently enlarged. The nose is generally dry, or discharges thick mucus in large quantity; and epistaxis occasionally occurs. The eye-lids are subject to chronic inflammation; and eruptions behind the ears and on the scalp, or other parts, are very frequent. Copious discharges of mucus, sometimes mixed with blood, take place from the bowels, and from the bronchial membrane; and are indeed common from all the mucous surfaces. The brain and spinal marrow frequently become the seat of secondary irritation, and hydrocephalus, epilepsy, and chorea, or paralysis may be the consequence; but the most frequent and most important of all the consequences of this disordered state of the digestive organs is tuberculous cachexia."

Our author, like his predecessors, has divided consumption into a variety of stages, and traced the symptoms, common to each, with much accuracy. He has also given the physical signs, peculiar to the various stages, which should be carefully studied. The first symptom of tubercular secretion in the lungs, is an occasional cough, especially in the morning, with the expectoration of a clear ropy saliva, which seems to escape from the posterier fauces. The breathing gradually becomes short, and somewhat difficult, the cough is more troublesome, especially when ascending elevations, the pulse is a little quick after meals or towards evening, with slight chilliness, followed by more or less thirst and heat, especially of the palms of the hands and soles of the feet. The febrile symptoms pass off by perspiration, which is more or less profuse as the disease advances.

At this period, the countenance is paler than usual, or changes its color with much rapidity, after the least exercise. There is also a general languor both of body and mind, with a harsh, dry skin, softness of the muscular system, and general emaciation. The lungs contain, at this time, more or less tuberculous matter, in a crude state. The process of softening has not yet commenced, and the pulmonary tissue is but little changed, even in the immediate vicinity of the morbid secretion,—facts which clearly prove, that the constitutional disturbance is not the result of the tuberculous deposit.

At this period, the physical signs of the disease are generally very obscure. Auscultation reveals but little to us unless the tuberculous deposit be very extensive, and even then it requires an experienced ear to decide on the true nature of the disease. As tubercles are almost always formed immediately under the clavicles, it is here that the stethescope will first reveal to us the confirmed respiratory murmur of the early stages of true phthisis.

We will not follow our author through his detail of the various symptoms peculiar to every stage of consumption. Suffice it to say that it is in this first stage alone that any thing is within the power of the physician. When the second, marked by a change in the expectoration, with increased cough, fever preceded by chills, and hurried respiration, commences, he can only paliate symptoms, and thus render the path of his patient to the grave as pleasant as the various circumstances will permit.

After giving the symptoms of acute or rapid consumption, which vary from those of the others, only in being more severe, and running their course with more rapidity, our author remarks, p. 36:

"The error into which this variety of acute phthisis is calculated to lead an inexperienced or careless practitioner, is that of considering and treating it as a purely inflammatory disease, and using more active measures, and giving a more favorable prognosis than the case justifies. It is true that inflammation in some part of the respiratory organs often exists, complicating the tuberculous disease; but it requires to be treated with much more delicacy than a simple inflammation, and a very different prognosis should be given. An inquiry into the previous health of the patient and careful observation of the symptoms will alone unveil the real nature of the disease."

Since pathological anatomy has received the special attention of the profession, consumption is found to be a frequent disease of infancy and childhood. In a single hospital in France, in which children alone are received, one of the physicians states that five sixths of those who die there are either cut off by consumption, or have tuberculous lungs. Our author devotes several pages to the consideration of this species of consumption. He infers that the tuberculous matter is almost always deposited in the bronchial glands, and hence it may occasionally be made to disappear, by a judicious treatment, as is sometimes the case in the lymphatic glands of the neck. This termination, however, is no doubt very unfrequent, and it is quite probable that the disease is almost, if not quite, as fatal as it is in later life. The writer of this review thinks he once succeeded in arresting a case of this kind, by the use of gentle emetics, cathartics and iodine, with slight irritation to the surface of the enlarged glands on the neck, but he is not sure that the disease is cured, although the child has enjoyed good health, *to all appearance*, for the last two years.

Our author next devotes about fifty pages to the consideration of the symptoms of tuberculous phthisis in detail, as well as the character of the various lesions apparent upon *post mortem* examinations. In the whole of which he has manifested much research and acute observation. He next proceeds to treat of its cure, when he mentions a few cases of recovery, principally drawn from the pages of Laennec and others, without attaching much importance to any of them. We think this is correct, for it is probable that these patients might have recovered without the use of medicine. He recommends the curative means to be directed to the general system, with but little reference to the local disease.

He next gives a number of diseases which attend and complicate pulmonary consumption. The most important of these are lesions of the abdominal mucous tissue, which we think form component parts of it. It is true, ulcerations of the epiglotis, larynx, trachea, &c., occur late in the disease, and may fairly be said to be its offspring, yet the irritation of the abdominal vicera, producing dyspepsia, diarrhœa, and bilious derangement, seem to precede in many instances, the tubercular deposit, and it is more than probable, that they all originate in a common state of the general system. As all of these, together with the fatty transformation of the liver and fistula in ano have been noticed by other writers, we shall proceed to notice his remarks on the duration of consumption, drawn from Louis and Bayle. From these it would seem, that the greatest proportion of deaths occur at from five to eight months after the attack, and that the mean duration of the disease is twenty-three months, although, in a few instances it may last nearly as many years. It also appears, that the two sexes among whites, are about equally liable to the disease, while among blacks, the proportion of deaths among females is increased to nearly one and a half per cent.

We shall pass over the various causes to which he attributes pulmonary consumption, by remarking, that he thinks it is generally hereditary, and proceed to notice his chapter on PREVENTION.

As he deems the disease hereditary, he thinks the first abuse necessary to be corrected, is in marriage. Families inclined to consumption should never intermarry; a wise proposition, it is true, but one not likely to be attended to, unless the parties relinquish their entire agency in the matter, to the doctor and the parson. Relatives should not intermarry, as such alliances evidently tend to aggravate hereditary predisposition, as well as to deteriorate the intellectual and physical powers of the entire generation. Pregnant females should also guard their healths in the most scrupulous manner, for it is more than probable, that let the physiological connexion between the mother and child be what it may, lesions may be commenced in utero, which will utimately destroy the child, or at least very much impair the energy of its constitution. After the birth of the child, the principal means of prevention may be sumned up under the heads of food, clothing, dress, bathing, air, exercise, and residence. We have neither time nor space to follow our author through what he has said under these heads.

We will now notice our author's remarks on the treatment of tuberculous cachexia, and then close our account of his work.

For the treatment of this constitutional affection, he relies principally on alteratives; such as mercury, iodine, antimony, taraxicum, sarsaparilla, and the alkalies. The most important of these, however, appears to be iodine; and hence we shall let him speak for himself on the subject. We do not see that he uses the others in any way different from his predecessors. On page 235 he observes:

"The action of iodine on the animal economy in a great degree resembles that of mercury. They both promote the excretory functions, and it is thus, most probably, that they increase the activity of the assimilative functions. Mercury is more quick in its operation than iodine, and we are better acquainted with its management; but the operation of iodine on the economy appears to be more general. Its action on the uterine system is decided; on the secreting functions of the liver and kidneys its operation is also evident; and it appears to promote the insensible perspiration. It thus diminishes abdominal plethora, promoting the activity of the eliminative functions, and, through them, of the assimilative. But in whatever we may attempt to explain its mode of operation, the beneficial effects of iodine on strumous constitutions are undoubted. Under its influence, when it is judiciously employed, the patient recovers flesh, strength and color; hitherto pale, relaxed, and feeble, he becomes full, strong, and florid; glandular swellings disappear or are greatly reduced; scrofulous ulcers heal; swellings of the joints are reduced, and the limbs restored to their natural proportions; and the condition of the whole animal economy is greatly improved."

This article, however, should never be employed when the bowels are irritable, or when there is great irritability or sensibility of the nervous system in general. After venesection, resinous and mercurial cathartics, and gentle narcotism, it may be used with much advantage, especially in *young* subjects. We have derived much benefit from its use in several instances. In one instance, a colored boy, five or six years of age, was effectually cured, to all appearance, after the glands about the neck had become very much enlarged. He continued its use for nearly eleven months. It was given in solution, with the hydriodate of potash in water, in the proportion of one-half of a grain of the former, and a grain of the latter to four ounces of distilled water. Its use was preceded by mercurial cathartics. When it produced unpleasant effects on the bowels, it was suspended for a few days, or accompanied by small portions of blue pill, in combination with hyosciamus or the salts of opium. Dr. L. C. Rives, in a former number of this Journal, speaks highly of its effects in scrophulous diseases. W. W.

